# Serum vascular endothelial growth factor load and interleukin-6 in cancer patients – reply

**DOI:** 10.1054/bjoc.1999.1253

**Published:** 2000-06

**Authors:** R Salgado, I Benoy, R Weytjens, P B Vermeulen, L Y Dirix

**Affiliations:** Angiogenesis Group, Oncology Center, St Augustinus Hospital Antwerpen, Oosterveldlaan 24, Wilrijk, B-2610, Belgium


DOI: 10.1054/ bjoc.1999.1253, available online at http://www.idealibrary.com on IDEAL®

### Serum vascular endothelial growth factor load and interleukin-6 in cancer patients – reply

**Sir**

We read with great interest the letter of O'Byrne and colleagues. They confirm previous observations on the relation between platelet count and serum vascular endothelial growth factor (VEGF) and on the predictive value of increased serum VEGF and poor outcome (Dirix et al, 1996, 1997; Salven et al, 1997). These data also suggest that platelets might be considered as mediators of VEGF induced tumour-angiogenesis, and thus influence tumour growth (Pinedo et al, 1998).

In a recent study we have examined the VEGF load in platelets ($VEGF^{PLT}$) from 25 healthy volunteers, 30 previously untreated patients with operable breast cancer and 70 patients with advanced breast cancer. The healthy volunteers had a VEGF load of 0.41 pg $10^{-6}$ platelets, which gradually increased to 1.00 pg $10^{-6}$ platelets for the non-metastatic group to 1.58 pg $10^{-6}$ platelets for the advanced group (Kruskal–Wallis test, $P < 0.0001$).

We have previously suggested that interleukin-6 (IL-6) might be particularly important in tumour-related angiogenesis (Salgado et al, 1999). Preliminary data derived from the same three subsets of patients confirm these data, showing a significant correlation between circulating levels of IL-6 and the $VEGF^{PLT}$ in the group with advanced breast cancer (Spearman rank test, $P < 0.002$). A significant difference was also observed between mean IL-6 levels in the control vs locoregional disease group (0.5 pg $ml^{-1}$ vs 1.4 pg $ml^{-1}$ respectively, Mann–Whitney *U*-test, $P = 0.041$) and between these and the advanced group (1.4 pg $ml^{-1}$ vs 12.4 pg $ml^{-1}$ respectively, Mann–Whitney *U*-test, $P < 0.0001$).

In a second study we measured serum IL-6 levels in the effluent mesenteric vein in seven patients suffering from an operable colorectal carcinoma and compared these values with those obtained from an arterial (art.) and peripheral venous (pv.) blood sample. Arterial and peripheral venous IL-6 did not reach the detection limit of the assay (3.1 pg $ml^{-1}$) in 75% and 63% of the patients respectively (means of 2.2 pg $ml^{-1}$ and 2.5 pg $ml^{-1}$ respectively), whereas 88% of the patients had high (> 3.1 pg $ml^{-1}$) venous mesenteric IL-6 levels (mean 15.7 pg $ml^{-1}$; Fisher exact test between art. vs vm. values, $P = 0.04$). Remarkably, in three other patients known to have disseminated disease, 67% had both peripheral venous and arterial IL-6 values (means of 7.7 pg $ml^{-1}$ and 7.22 pg $ml^{-1}$ respectively) higher than the detection limit. All the patients had high mesenteric venous values (mean 38 pg $ml^{-1}$, Fisher exact test between art./pv vs vm. values, $P = 1.00$). These data indicate that both the primary tumour and its metastases are sites of IL-6 production. In primary renal cell carcinoma similar observations have been made (Blay et al, 1994).

Tumour growth might be influenced by IL-6 at different levels. First, IL-6 might induce tumour cell proliferation. Second, IL-6 can stimulate VEGF expression in tumour cells, thus contributing locally to a pro-angiogenic environment. Third, IL-6 induces fibrinogen secretion by hepatocytes. As fibrinogen extravasation kinetics are enhanced in tumours, IL-6 might in this manner contribute to the formation of a fibrin matrix which is considered to be important in stromal remodelling, a process intrinsically related to angiogenesis. Fourth, circulating endothelial cell progenitors (ECP) might contribute to intra-tumoural angiogenesis. As these ECP originate from bone marrow, IL-6 might influence their extravasation. As these ECP contain the VEGF-receptor, enabling them to differentiate into endothelial cells, they may be an additional stimulus for ongoing intra-tumoural angiogenesis. Fifth, our previous results confirm the thrombopoietic properties of IL-6 and suggest a role of IL-6 in the VEGF-regulation in platelet precursors, the megakaryocytes. This might explain in part the higher VEGF load in platelets from advanced cancer patients. Platelets could subsequently adhere, aggregate and release this higher VEGF load in tumours.

These observations might explain in part why patients with high IL-6 levels, high platelet counts, high VEGF levels and, as demonstrated by O'Byrne et al, a high $VEGF^{PLT}$ have worse prognosis. The recent data provided by O'Byrne and colleagues provide further evidence that platelets may be considered as mediators of VEGF mediated endocrine modulation of tumour-related angiogenesis.

*R Salgado, I Benoy, R Weytjens, PB Vermeulen, LY Dirix*
*Angiogenesis Group, Oncology Center, St Augustinus Hospital Antwerpen, Oosterveldlaan 24, B-2610 Wilrijk, Belgium*

**REFERENCES**

Blay J-Y, Schemann S and Favrot MC (1994) Local production of interleukin 6 by renal adenocarcinoma in vivo. *J Natl Cancer Inst* **86**: 238

Dirix LY, Vermeulen PB, Hubens G, Benoy I, Martin M, De Pooter C and Van Oosterom AT (1996) Serum basic fibroblast growth factor and vascular endothelial growth factor and tumour growth kinetics in advanced colorectal carcinoma. *Ann Oncol* **7**: 843–848

Dirix LY, Vermeulen PB, Pawinsky A, Prové A, Benoy I, De Pooter C, Martin M and Van Oosterom AT (1997) Elevated levels of the angiogenic cytokines basic fibroblast growth factor and vascular endothelial growth factor in sera of cancer patients. *Br J Cancer* **76**: 238–243

Pinedo HM, Verheul HMW, D'Amato RJ and Folkman J (1998) Involvement of platelets in tumour angiogenesis? *Lancet* **352**: 1775–1777

Salven P, Teerenhovi L and Joensuu H (1997) A high pretreatment serum vascular endothelial growth factor concentration is associated with poor outcome in non-Hodgkin's lymphoma. *Blood* **90**: 3167–3172

Salgado R, Vermeulen PB, Benoy I, Weytjens R, Huget P, Van Marck E and Dirix LY (1999) Platelet number and interleukin-6 correlate with VEGF but not with bFGF serum levels of advanced cancer patients. *Br J Cancer* **80**: 892–897

 © 2000 Cancer Research Campaign

